# Stay Present with Your Phone: A Systematic Review and Standardized Rating of Mindfulness Apps in European App Stores

**DOI:** 10.1007/s12529-020-09944-y

**Published:** 2020-11-20

**Authors:** Dana Schultchen, Yannik Terhorst, Tanja Holderied, Michael Stach, Eva-Maria Messner, Harald Baumeister, Lasse B. Sander

**Affiliations:** 1 Clinical and Health Psychology, Institute of Psychology and Education, Ulm University, Albert-Einstein-Allee 41, Ulm, Germany; 2 Research Methods, Institute of Psychology and Education, Ulm University, Albert-Einstein-Allee 47, Ulm, Germany; 3 Clinical Psychology and Psychotherapy, Institute of Psychology and Education, Ulm University, Albert-Einstein-Allee 47, Ulm, Germany; 4 Institute of Databases and Information Systems, Ulm University, James-Franck-Ring, 89081 Ulm, Germany; 5 Institute of Psychology, Department of Rehabilitation Psychology and Psychotherapy, Albert-Ludwigs-University of Freiburg, Engelbersger Str. 41, 79085 Freiburg, Germany

**Keywords:** Mindfulness, Apps, MARS, Mobile health, mHealth, Systematic review

## Abstract

**Background:**

Mindfulness-based interventions show positive effects on physical and mental health. For a better integration of mindfulness techniques in daily life, the use of apps may be promising. However, only a few studies have examined the quality of mindfulness apps using a validated standardized instrument. This review aims to evaluate the content, quality, and privacy features of mindfulness-focused apps from European commercial app stores.

**Methods:**

An automated search engine (webcrawler) was used to identify mindfulness-focused apps in the European Apple App- and Google Play store. Content, quality, and privacy features were evaluated by two independent reviewers using the Mobile Application Rating Scale (MARS). The MARS assesses the subscales engagement, functionality, aesthetics, and information quality.

**Results:**

Out of 605 identified apps, 192 met the inclusion criteria. The overall quality was moderate (*M* = 3.66, *SD* = 0.48). Seven apps were tested in a randomized controlled trial (RCT). Most of the apps showed a lack of data security and no privacy policy. The five apps with the highest ratings are from a credible source, include a privacy policy, and are also based on standardized mindfulness and behavior change techniques.

**Conclusions:**

The plethora of often low-quality apps in commercial app stores makes it difficult for users to identify a suitable app. Above that, the lack of scientific verification of effectiveness and shortcomings in privacy protection and security poses potential risks. So far, the potential of mindfulness-focused apps is not exploited in commercial app stores.

## Introduction

Mindfulness is described as an approach to be aware and attentive of the present moment in an open and accepting way without any judgment or criticism [1–3]. In recent years, research interest in mindfulness techniques is growing [4–6]. Previous studies investigating the efficacy of mindfulness interventions showed positive effects on physical and mental health (e.g., depression, anxiety, well-being) in clinical and non-clinical populations [7–13]. Various mindfulness techniques are used in health promotion and clinical practice, including standardized programs like mindfulness-based stress reduction (MBSR) and mindfulness-based cognitive therapy (MBCT; e.g., [14–17]). The techniques are instructed by an experienced trainer and practiced in a weekly group session [3, 18]. Furthermore, individual elements of mindfulness techniques like meditations (sitting/movement), body scan, mindfulness yoga or breathing exercises, and visualization exercises have found their way into a variety of different treatments [3, 19]. Studies repeatedly pointed out that it is important for the success of interventions to integrate techniques into daily life [20]. However, this involves the challenge of health behavior change [20, 21]. The use of apps has repeatedly been discussed to overcome these challenges [21–24]. Apps can be used flexibly in terms of time and place, are readily available to a broad audience, and offer reminder functions, and supporting material (e.g., pictures and videos) can have a positive impact on behavioral change [25–28].

Besides these advantages, there are also some limitations in the use of health apps, which include inadequate protection of data and privacy features and the lack of an informed consent [29–35]. Furthermore, only a few studies investigating the efficacy of mindfulness apps [36–41] or other health-related conditions such as depression, anxiety, pain, and posttraumatic stress disorders have been published [42–47]. Another disadvantage are missing international quality standards for the development of apps, as well as the lack of integration of healthcare providers in the development process [26, 48–51]. In a previous review, Mani and colleagues [36] reviewed 23 mindfulness apps from an Australian app stores. The results of this study indicated that the median score exceeds an acceptable score and that quality could be improved. However, this review is limited through the small number of included apps, the inclusion of apps only from the Apple app store, and the use of a single search term (“mindfulness”) focusing on the Australian app store.

Hence, the aim in this study was to conduct a systematic review and quality rating of mindfulness apps in the European commercial app stores and expand the search to different app stores and to use a comprehensive search term and engine. Moreover, this review also included a rating on general app characteristics such as privacy policy, techniques used, and content features, as well as the specific examination of the relationship between user and quality ratings. The five best-rated apps are described in detail to give users and healthcare providers an overview of possible applications of mindfulness apps in practice.

## Method

### Search Strategy and Procedure

Using an automated search engine (webcrawler), a search in the European app stores from Apple App and Google Play store on the 24th of October 2018 was conducted. Therefore, the following keywords were used: “mindfulness,” “meditation,” “yoga,” and “body scan.”

All apps were listed in a central database, and duplicates were automatically removed. Apps from the Apple App and Google Play store were screened against the following inclusion criteria: (1) sufficient content and conceptualized for mindfulness (i.e., exclusion of fitness and yoga apps or in case that apps only provide timer, reminder, music or quotes), (2) provided in German or English language (in accordance with the reviewers’ language skills), and (3) officially available in the European Apple App or Google Play store. In a second step, apps were downloaded and checked regarding the aforementioned criteria and, if the app was fully functional for the review (e.g., no device problems, development/testing phase).

### Data Extraction, Evaluation Criteria, and Instruments

Two independent reviewers evaluated each app using the German version of the Mobile Application Rating Scale (MARS) [52]. All reviewers had a psychology degree and scientific background. Before starting with the evaluating process, every reviewer received a free online training [https://www.youtube.com/watch?v=5vwMiCWC0Sc]. To evaluate the apps, every reviewer had to test each app for at least 15–20 min with the focus on the different subscales of MARS: user engagement, functionality, visual aesthetics, and information quality.

The interrater reliability (IRR) between the two reviewers was calculated. The excellent intra-class correlation of ≥ 0.75 indicates a satisfactory score [53]. In case that the score of an app-rating was below this score, a third rater (DS or LS) was consulted. To combine ratings of each app, averaged mean scores of both reviewers were used for each subscale as well as an overall score.

### App Characteristic and Quality Rating: MARS-German

To describe the app characteristics, different descriptive data was obtained, including (1) app name, (2) platform (Apple App or Google Play store), (3) content-related subcategories, (4) price, (5) goals, (6) methods, (7) data protection and privacy, (8) user rating from the Apple App and Google Play store, and (9) number of conducted randomized controlled trials. It should be noted that this part was slightly reduced to focus on the crucial mindfulness information.

Additionally, privacy and security features were reviewed according to the MARS classification system on a 5-point scale (1 = inadequate; 2 = poor; 3 = acceptable; 4 = good; and 5 = excellent). The MARS quality rating comprises the four main subscales: (1) user engagement (5 items: entertainment, interest, customization, interactivity, target group), (2) functionality (4 items: performance, usability, navigation, gestural design), (3) aesthetics (3 items: layout, graphics, visual appeal), (4) information quality (7 items: accuracy of app description, goals, quality of information, quantity of information, quality of visual information, credibility, evidence base). For each subscale, a mean score was calculated [54]. Both review ratings averaged this score. Previous research evaluating psychometric criteria showed that reliability and objectivity of the MARS and the German version of the MARS were good to excellent [55, 56]. Additionally, Terhorst and colleagues [55] confirmed a good construct validity for the MARS.

## Results

### Search and App Characteristics

**Fig. 1:**
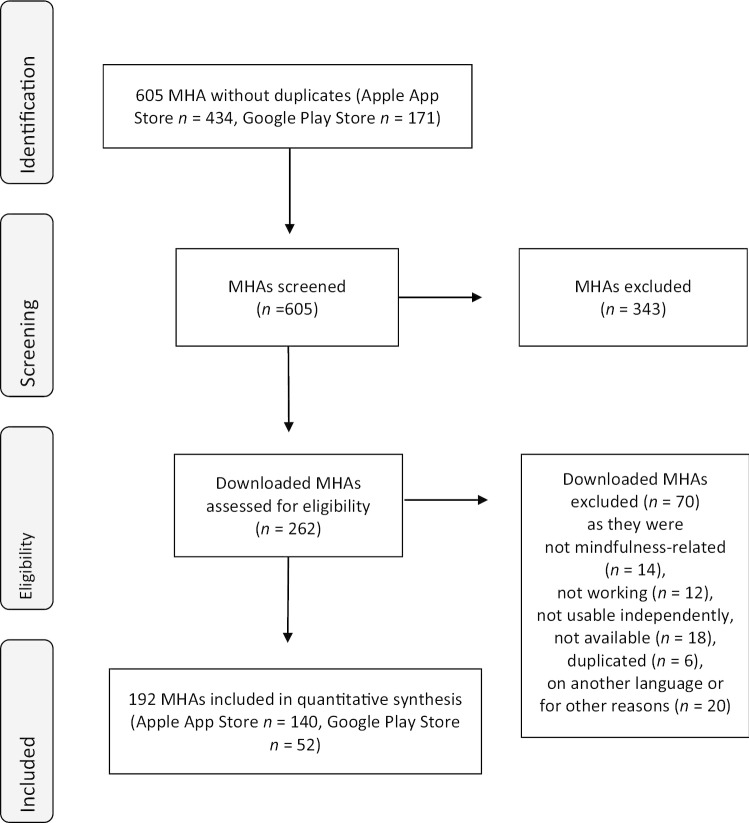
Flowchart of the inclusion process

The search resulted in 605 apps (Apple App store = 434, Google Play store = 171), of which 192 apps (123 English, 69 German) could be included (see Fig. 1).

### General Characteristics

Most of the apps were free of charge (*n* = 157, 82%). The prices for the remaining apps ranged between 0.99 EUR and 9.99 EUR (*M* = 3.49 EUR; *SD* = 2.03). Most of the apps aimed to improve well-being (*n* = 175, 91%) and reduce stress (*n* = 144, 75%), followed by improvement of physical health (*n* = 58, 30%), reduction of anxiety (*n* = 55, 29%) and depressive symptoms (*n* = 23, 12%), emotion regulation (*n* = 26, 13%), support for behavioral change (*n* = 19, 10%), entertainment (*n* = 12, 6%), improvement of social behavior (*n* = 12, 6%), as well as reduction of addictive behavior (*n* = 2, 1%). Sixty apps (31%) were classified with other goals (e.g., information about mindfulness, improving sleep quality, self-awareness and concentration).

### Data Security and Privacy Policy

Data security and privacy features showed that 15 apps (8%) were password-protected, 14 apps (7%) offered the possibility to use a login-area, and 44 apps (23%) included a privacy policy (7 apps (4%) active confirmation, 12 apps (6%) passive privacy policy). Sixty-four apps (24%) gave information about contact details and the imprint. Four apps (2%) offered an emergency function, including helpline numbers or addresses for medical assistance.

### App Quality Rating

**Fig. 2:**
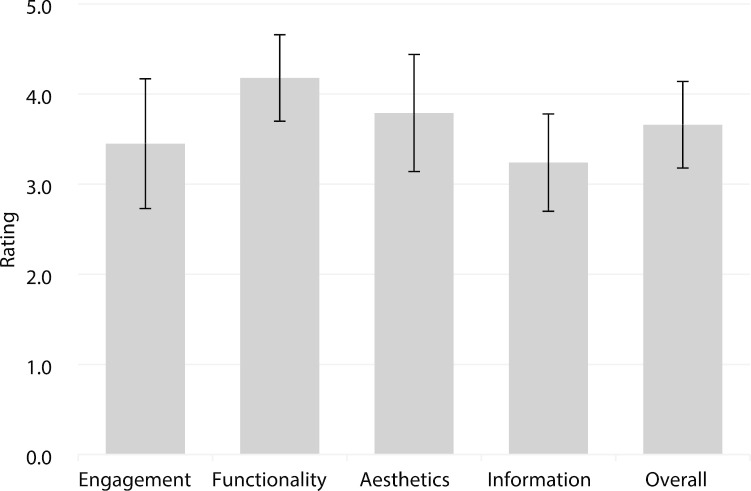
Quality of included mindfulness-focused apps rated with the Mobile Application Rating Scale (1, inadequate; 2, poor; 3, acceptable; 4, good; and 5, excellent)

The intra-class correlation, indicating agreement of both raters, was excellent (ICC = 0.87, 95% CI: 0.86 to 0.88). The average overall quality rating for mindfulness apps was *M* = 3.66 (*SD* = 0.48, range 2.47–4.75), demonstrating a moderate quality. For the different subscales, the following average ratings were found: engagement (*M* = 3.45, *SD* = 0.72, range 1.60–5.00), functionality (*M* = 4.18, *SD* = 0.48, range 3.00–5.00), aesthetics (*M* = 3.79, *SD* = 0.65, range 1.66–5.00) and information (*M* = 3.24, *SD* = 0.5, range 2.00–4.58). All data are summarized in Fig. 2.

### Evidence-Based Ratings and Methods

Only seven apps (4%) were tested in randomized controlled trials (RCTs), including “Mindfulness Coach,” “Pacifica for Stress & Anxiety,” “Headspace,” “Calm: Meditation and Sleep,” “7Mind Meditation & Mindfulness,” “Smiling Mind,” and the German app “Die Achtsamkeit App” (translation: “The mindfulness app”; e.g., 36–41, 57–59). Fourteen apps were investigated regarding different other variables such as usability and satisfaction in non-RCT or qualitative assessments [60–65]. The mindfulness applications offered various standardized methods, including relaxation (*n* = 153, 80%), breathing (*n* = 118, 62%) and body exercises (*n* = 59, 31%), mindfulness (*n* = 117, 61%), acceptance (*n* = 28, 15%), and resource management (*n* = 4, 2%). Furthermore, different apps focused on monitoring and tracking (*n* = 55, 29%), feedback (*n* = 19, 10%), information and education (*n* = 74, 39%), advices (*n* = 46, 24%), and training (*n* = 42, 22%).

### User vs. Expert Rating

For 161 apps a user-star-rating (*M* = 4.31, *SD* = 0.60) could identified, while 31 apps were not rated by the users. Analyses resulted in correlations between user-star-ratings and the MARS subscales engagement (*r* = 0.221, *p* = 0.005), functionality (*r* = 0.158, *p* = 0.046) as well as aesthetics (*r* = 0.202, *p* = 0.010). No correlation could be found between user rating and the overall score as well as information quality (*p*s > 0.31).

### Features of the Five Top-Rated Apps

In the following, the five best-rated apps (range of the overall quality score 4.61–4.75) will be presented. Besides the high overall score (> 4.61), all apps are from a credible source (i.e., government, university, non-profit organization, or specialized company) and contained a data privacy statement. Four of them also provided contact information on the imprint, and three of them had an emergency function. Two apps are asking for challenges in daily life (e.g., anxiety, motivation, exam stress, loss, loneliness) to create an individual program. Further, three apps offered guided support, whereas the app “Wysa Anxiety & Depression Bot” also provided professional guidance by a therapist for 29.99 EUR per week. This guidance includes two live chat sessions per week and unlimited messaging support. All apps presented psychoeducation and information (e.g., emotion perception and regulation, coping skills, problem solving, goals, gratitude, mindfulness techniques) via text and/or audio. Two apps included reminder functions for daily practice. Four apps were complemented by tracker or journal functions with the focus on daily practice, but also mood and symptoms could be tracked. Not surprisingly, breathing and relaxation exercises (e.g., PMR, meditation, body scan) were integrated into all apps. All features are displayed in Table 1.

**Table 1:** Features of the five best-rated apps

App name	Credible source	Privacy policy	Contact information	Emergency functions	Individual program	Guided support	Psychoeducation/information	Reminder	Tracker	MBSR/MBCT-based practices^a^
Wysa Anxiety and Depression Bot	✔	✔	✔	✔	✔	✔	✔	✔	✔	✔
Youper—AI Assistant	✔	✔	✔	x	✔	✔	✔	x	✔	✔
Relax Now: Hypnosis Meditation	✔	✔	✔	x	x	✔	✔	x	x	✔
Mind the Bump—A Mindfulness Meditation tool for new and expecting parents	✔	✔	✔	✔	x	x	✔	✔	✔	✔
Mindfulness Coach	✔	✔	x	✔	x	x	✔	x	✔	✔

^a^Body scan meditations, breathing meditations, walking meditations, sitting meditations

## Discussion

In this study, the quality of 192 mindfulness-focused apps in the European commercial app stores using a standardized rating instrument was systematically reviewed. Additionally, the current evidence base as well as privacy and security features on the mindfulness-focused apps were evaluated. Furthermore, the content and features of the five best-rated apps were displayed. Given the plethora of available apps, users and healthcare providers might have problems to identify an app that suits their needs. Shortcomings in privacy and security, the mostly lacking scientific evidence-base, lack in the information quality pose additional constraints.

Only seven apps (4%) provided evidence on the effectiveness of the reviewed apps [39, 57–59]. This shortcoming in the scientific evaluation of apps is in accordance with the review of Mani and colleagues [36] and other health-related app reviews [26, 43–48]. This might partly be explained by the fast development cycle of apps and continuous improvement through user feedback, which does not fit with the time requirements of current scientific studies [66–68]. Consequently, scientific- and technology-based developed interventions may be out of date at the time when they are validated. Mohr and colleagues [69] suggest a solution to this problem by creating a continuous evaluation of behavioral intervention technologies (CEEBIT) through systematic prospective analyses.

Another concern was that the MARS subscale “information quality” implies the most deficits given the lowest average score of all subscales. Only 18 of the 192 included mindfulness-focused apps had a score higher than 4.0, which would define the app as a high-quality app on the “information quality” subscale. To prevent misinformation and adverse effects of mindfulness app use in the future, information quality must be improved. Furthermore, the user rating did not correlate with the subscale “information quality,” but with engagement, functionality, and aesthetics. However, wrong or misleading information could result in a decrease in users’ safety [46]. The involvement of experts in the development process (e.g., psychotherapists, researchers, and users) might help to overcome this problem. Moreover, a better description of the app on the app store website, a definition of the specific goals and better content (i.e., general information to mindfulness and how to use the different techniques) would improve information quality.

In addition to the risk of misinformation, users are also at risk from the described deficits in terms of data protection and data security. This is in accordance with prior studies [45, 46, 70]. In the case of violated security approaches, careful use of these apps is proposed [71]. Another issue is that some apps are also submitted private data to commercial entities without any permission [34, 35]. Only a few apps (2%) are offering helpline numbers or points of assistance in case of an emergency. However, there should be at least a disclaimer that in case of unexpected symptoms, users should be searching for professional help or how to access other treatment options, mainly because most mindfulness app users suffer from stress [72–74] as well as anxious and depressive symptoms [9].

Despite the named issues, the average MARS score indicated an average overall quality in line with the results of Mani and colleagues [36] who rated 23 apps in the Australian Apple App store in 2015. However, whereas only one app could be identified as a high-quality app (overall mean score > 4.0) in the study of Mani and colleagues [36], the present study identified 50 apps above the MARS overall mean score of 4.0. The five top-rated apps described in detail contained a range of high-quality content and features that can facilitate the application of mindfulness techniques in practice. Furthermore, these apps also include additional functions such as monitoring and tracking, feedback and (guided) support, and education as well as advice, which might foster behavior change.

Based on the positive and critical aspects of the different mindfulness-focused apps, it can be hard to find a suitable app. Consequently, there is a need for independent information platforms (e.g., German Mobile Health App Database: http://mhad.science/; PsyberGuide: https://psyberguide.org/) offering reliable information for health care providers and health seekers about the app content and quality, which could be combined by user and health expert ratings. Moreover, to protect customers and improve app quality, there is an urgent need for universal guidelines regarding (mental health) app control [75, 76]. Therefore, the focus should be on app evaluation, validation, and quality assessment in which health care professionals, policy makers, user, and developers should be involved before launching an app.

This study has several limitations. Firstly, the app market is rapidly growing [69, 77], and the rated apps of this review can be revised. Consequently, a new search and rating process could lead to different apps as well as other evaluations. Secondly, the apps could no longer be available, or due to the focus on the Apple App and Google Play store, apps from other stores could be overlooked due to the selection bias. However, it should be noted that both app stores cover 99% of the total market [78]. Consequently, the missed number of apps should be low. Thirdly, the rated mindfulness apps are limited to specific searches. Fourthly, only German and English apps were included in the rating process due to the language skills of the reviewer. Accordingly, it is not guaranteed that these apps are also available in other countries. Lastly, this review is only based on the MARS rating, which could be extended by different quality rating scales such as ENLIGHT [79] and the APA App Evaluation [80, 81].

## Conclusion

In this comprehensive investigation of 192 apps focusing on mindfulness, an average overall quality was found, resulting in possible risks for the users due to a lack of information and content quality, missing privacy policy, and data security as well as lack of evidence base. To offer reliable information for healthcare providers and health seekers, independent platforms, such as the German Mobile Health App Database or PsyberGuide, are needed. In general, apps have a high potential to reach a broad audience who aims to engage in mindfulness practice in daily life and to improve different mental health variables (e.g., stress, depressive and anxiety symptoms). However, at the moment, the commercial distribution channels fail to unfold this potential.
